# Understanding Ehlers-Danlos Syndrome: A Claims-Based Analysis of Healthcare Cost Patterns

**DOI:** 10.36469/001c.145941

**Published:** 2026-07-15

**Authors:** Samson A. Oyediran, Youngran Kim, Zhanna Novikov, Trudy M. Krause

**Affiliations:** 1 The University of Texas Health Science Center at Houston, Texas; 2 UTHealth School of Public Health Center for Health Care Data University of Texas Health Science Center at Houston

**Keywords:** Ehlers-Danlos syndrome, healthcare costs, claims database, rare disease, economic burden, outcomes research

## Abstract

**Background:** Ehlers-Danlos Syndrome (EDS) is a rare connective tissue disorder associated with substantial clinical complexity and healthcare utilization. Despite increasing recognition of EDS, little is known about its economic burden in the United States. **Objective:** To assess the all-cause healthcare costs for individuals with newly diagnosed EDS during the pre-diagnosis year and the first 2 years post-diagnosis. **Methods:** A retrospective cohort study was conducted using the Merative™ MarketScan® Commercial Claims and Encounters Database (2016-2022). Newly diagnosed patients were identified using ICD-10-CM codes, with the first observed diagnosis defined as the index date. Continuous enrollment for 12 months pre-index and 24 months post-index was required. Costs represented gross payments to providers, adjusted to 2022 US dollars. Outcomes included inpatient, outpatient, and pharmacy expenditures, with outpatient services further stratified. Two-part models were estimated: a logistic regression for the probability of incurring costs and a GLM gamma model with log link for cost intensity among users. **Results:** The study included 4765 patients (mean age, 28.8 years; 78% female). Total annual costs rose from 19 733pre−indexto28 922 in the first year post-diagnosis, then declined to $21 804 in the second year, though remaining above baseline. Outpatient and pharmacy services were the largest contributors, while inpatient admissions, though less frequent, were resource intensive when they occurred. Two-part models showed that the likelihood of incurring any cost was significantly higher in Year 1 (β = 4.15, P < .001) but not in Year 2 (β = –0.03, P = .845). Among cost users, expenditures remained elevated in both Year 1 (β = 0.41, P < .001) and Year 2 (β = 0.13, P < .001). Female sex and higher Charlson Comorbidity Index scores were associated with significantly greater costs. Costs peaked immediately after diagnosis, driven by outpatient and pharmacy use, and remained elevated in Year 2. Patient characteristics, particularly comorbidities, strongly influenced expenditures. **Conclusions:** Healthcare costs for patients with EDS peaked during the first year following diagnosis and remained elevated in the second year relative to pre-diagnosis levels. These findings highlight the substantial and persistent economic impact of EDS on the healthcare system.

## INTRODUCTION

Ehlers-Danlos syndrome (EDS) is a heterogeneous group of hereditary connective tissue disorders caused by different genetic defects in collagen. Collagen is one of the major structural components of the body. Collagen is a tough, fibrous protein and serves as a building block essential in both strengthening connective tissue (eg, bones) and providing flexibility where needed (eg, cartilage).^1^ These disorders primarily involve the skin, muscles, skeleton, joints, and blood vessels. It is characterized by hypermobility of the joints, skin hyperextensibility, and tissue fragility.^1,2^

According to the National Organization for Rare Disorders,^3^ EDS has been classified into 13 subtypes based on clinical and genetic criteria, with the most common subtypes being hypermobility (hEDS), classical (cEDS), and vascular (vEDS) types. This classification not only assigns descriptive names to each EDS type but also abbreviates them using lowercase letters, aiding in distinguishing among the various types. For instance, hypermobile Ehlers-Danlos syndrome is now abbreviated as hEDS.^2^ Each type of EDS has its own set of features with distinct diagnostic criteria. Some features are seen across all types of EDS, including joint hypermobility, skin hyperextensibility, and tissue fragility.^2^

The overall prevalence of EDS is generally estimated to affect 1 in 5000 to 1 in 40 000 individuals; however, this varies significantly based on the EDS subtype.^1,4,5^ As more is learned about EDS, the hypermobility type (hEDS) has been found to be the most common, accounting for up to 90% of cases. Furthermore, the lack of standardized diagnostic criteria and the clinical heterogeneity of EDS contribute to the difficulty of determining its prevalence accurately.^2^ Patients often present with multisystem involvement, requiring frequent and diverse medical interventions. The complexity of disease management suggests a potentially significant healthcare burden. However, the economic impact of EDS remains underexplored.

Existing literature on rare and chronic genetic disorders provides a useful framework for understanding potential cost trajectories in EDS. Studies in conditions such as cystic fibrosis, Duchenne muscular dystrophy, and hemophilia have shown that healthcare costs often surge during the initial diagnostic or early management period and subsequently stabilize as treatment becomes routine.^6,7^ These patterns reflect the intensity of diagnostic testing, specialist involvement, and long-term management common to chronic heritable diseases. Yet, little is known about whether this pattern holds true for EDS. Understanding cost trajectories and utilization trends is critical for planning clinical management and resource allocation.

The objective of this study was to assess the all-cause healthcare costs for individuals with newly diagnosed EDS during the pre-diagnosis year and the first 2 years post-diagnosis, using a large administrative claims database. Specifically, we aimed to quantify cost trajectories, identify major cost drivers, and assess demographic and clinical predictors of expenditures.

## METHODS

This retrospective cohort study employed a pre-post design using the Merative™ MarketScan® Commercial Claims and Encounters Database spanning 2016 through 2022. The analysis included individuals younger than 64 years of age with at least 1 confirmed diagnosis of EDS identified by *International Classification of Diseases, Tenth Revision* (ICD-10) codes Q79.60-Q79.69 (**Supplementary Table S1**). Because the analytic period encompassed years preceding the 2019 detail-level revision of EDS coding, all EDS subtypes were analyzed collectively; subtype identification before 2019 could not be reliably distinguished in the data.

**Figure 1.**
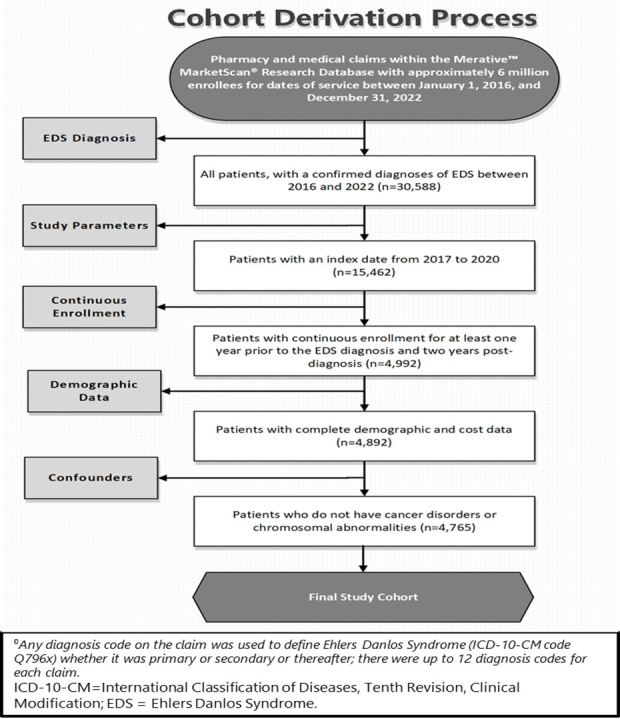
Cohort Derivation

The index date was defined as the first recorded EDS diagnosis between 2017 and 2020. To ensure continuous observation, eligible patients were required to have at least 12 months of enrollment before the index date (baseline) and 24 months after (follow-up), resulting in observation windows of 3 consecutive years per individual (**Table 1**). Patients were excluded if they had any malignancy or chromosomal abnormality (**Supplementary Table S2**), because these conditions introduce substantial variability in healthcare use and cost unrelated to EDS. Cases first identified in 2016 were also excluded to avoid misclassification of prevalent rather than incident diagnoses. Cohort selection procedures are summarized in **Figure 1**.

**Table 1.** Continuous Enrollment Periods

**Index Year**	**Pre-Diagnosis Period**	**Post-Diagnosis Period**
2017	01/01/2016–12/31/2017	01/01/2017–12/31/2019
2018	01/01/2017–12/31/2018	01/01/2018–12/31/2020
2019	01/01/2018–12/31/2019	01/01/2019–12/31/2021
2020	01/01/2019–12/31/2020	01/01/2020–12/31/2022

Demographic variables captured from enrollment files included sex, year of birth, and US region of residence. These characteristics enabled subgroup analyses across age, sex, and geography to better describe the distribution of EDS patients in the commercially insured population. The MarketScan® database has been widely used in health economics and outcomes research to evaluate utilization patterns, treatment costs, and patient outcomes across a variety of chronic conditions.^8–10^

Healthcare expenditures reflected total gross payments to providers prior to application of deductibles, copayments, or coordination-of-benefit adjustments. All costs were adjusted to 2022 US dollars using the Medical Care component of the Consumer Price Index.^11^ Costs were classified into inpatient, outpatient, and pharmacy categories. Outpatient costs were further divided into 11 subtypes: emergency department, primary care, specialty care, mental-health services, physical/occupational/speech therapy, diagnostics, laboratory, radiology, outpatient drugs, outpatient procedures, and ancillary or other services. Utilization was measured per patient per year and included inpatient admissions, length of stay, outpatient and emergency visits, provider encounters (primary, specialty, and non-physician), and prescription fills.

To estimate cost and utilization outcomes, two-part models were used.^12^ The first part modeled the probability of incurring any cost with a logistic regression (binomial family, logit link). The second part modeled cost intensity among users via a generalized linear model (gamma distribution, log link) for continuous cost outcomes and a negative-binomial specification for count-based utilization.^13,14^ Model covariates included study period (Year 1 and Year 2 post-index vs baseline), age group, sex, US region, and Charlson Comorbidity Index (CCI) category. The CCI, widely validated for claims-based comorbidity assessment,^12,15^ was calculated for each patient and categorized as 0 (no comorbidity), 1-2 (mild), 3-4 (moderate), or ≥5 (severe). Regression results are reported as coefficients with 95% confidence intervals.

All code lists (ICD-10-CM, CPT, HCPCS), comorbidity mappings, cost-classification algorithms, and model equations are provided in the **Supplementary Online Material**.

## RESULTS

### Study Population

The study population consisted of 4765 individuals observed over 4 years (2017-2020). Baseline characteristics of these samples are described in **Table 2**. The mean age was 28.8 years (SD = 15.5), with 78% female. Most patients were enrolled in preferred provider organization health plans (50%), followed by consumer-driven health plans (14%) and high-deductible health plans (15%). Regional distribution showed the largest share residing in the South (38%), followed by North Central (29%), West (17%), and Northeast (15%) regions. With respect to comorbidity burden, 61% of patients had no comorbidities, 35% had mild, 3.5% had moderate, and 1.1% had severe conditions, as measured by the CCI.

**Table 2.** Baseline Characteristics of EDS Patients by Index Year^a^

	**Total (N = 4765)**	**2017 (N = 1344)**	**2018 (N = 1130)**	**2019 (N = 1268)**	**2020 (N = 1023)**	***P* Value**
Age group						
0-17 y	1270 (27)	415 (31)	313 (28)	300 (24)	242 (24)	.003
18-34 y	1731 (36)	451 (34)	412 (36)	471 (37)	397 (39)	
35-44 y	814 (17)	205 (15)	192 (17)	230 (18)	187 (18)	
45-54 y	610 (13)	181 (13)	140 (12)	164 (13)	125 (12)	
55-64 y	340 (7.1)	92 (6.8)	73 (6.5)	103 (8.1)	72 (7)	
Sex						
Male	1028 (22)	321 (24)	255 (23)	262 (21)	190 (19)	.012
Female	3737 (78)	1023 (76)	875 (77)	1006 (79)	833 (81)	
Employee classification of subscriber						
Salary non-union	1399 (29)	381 (28)	340 (30)	383 (30)	295 (29)	<.001
Salary union	119 (2.5)	22 (1.6)	32 (2.8)	34 (2.7)	31 (3)	
Salary other	346 (7.3)	96 (7.1)	96 (8.5)	90 (7.1)	64 (6.3)	
Hourly non-union	518 (11)	130 (9.7)	131 (12)	141 (11)	116 (11)	
Hourly union	390 (8.2)	138 (10)	88 (7.8)	89 (7)	75 (7.3)	
Hourly other	183 (3.8)	53 (3.9)	38 (3.4)	52 (4.1)	40 (3.9)	
Non-union	561 (12)	207 (15)	113 (10)	117 (9.2)	124 (12)	
Union	248 (5.2)	95 (7.1)	69 (6.1)	50 (3.9)	34 (3.3)	
Unknown	1001 (21)	222 (17)	223 (20)	312 (25)	244 (24)	
Employee status						
Active full-time	4057 (85)	1139 (85)	940 (83)	1085 (86)	893 (87)	.220
Active part-time or seasonal	70 (1.5)	20 (1.5)	20 (1.8)	25 (2)	5 (.49)	
Early retiree	135 (2.8)	39 (2.9)	40 (3.5)	35 (2.8)	21 (2.1)	
Medicare-eligible retiree	21 (.44)	6 (.45)	6 (.53)	4 (.32)	5 (.49)	
Retiree (status unknown)	1 (2.1e^-02^)	1 (7.4e^-02^)	0 (0)	0 (0)	0 (0)	
COBRA continuee	3 (6.3e^-02^)	2 (.15)	0 (0)	1 (7.9e^-02^)	0 (0)	
Long-term disability	10 (.21)	2 (.15)	1 (8.8e^-02^)	5 (.39)	2 (.2)	
Surviving spouse/dependent	4 (8.4e^-02^)	1 (7.4e^-02^)	0 (0)	2 (.16)	1 (9.8e^-02^)	
Other/unknown	464 (9.7)	134 (10)	123 (11)	111 (8.8)	96 (9.4)	
Plan type						
Comprehensive	125 (2.7)	45 (3.4)	33 (3)	18 (1.4)	29 (2.9)	<.001
Exclusive provider organization	24 (.51)	7 (.53)	4 (.36)	8 (.63)	5 (.49)	
Health maintenance organization	616 (13)	167 (13)	125 (11)	187 (15)	137 (14)	
Point of service	218 (4.6)	53 (4)	43 (3.9)	68 (5.4)	54 (5.3)	
Preferred provider organization	2354 (50)	686 (52)	585 (53)	605 (48)	478 (47)	
Point of service with capitation	17 (.36)	12 (.9)	1 (9.1e^-02^)	1 (7.9e^-02^)	3 (.3)	
Consumer-driven health plan	664 (14)	199 (15)	148 (13)	174 (14)	143 (14)	
High-deductible health plan	689 (15)	162 (12)	163 (15)	202 (16)	162 (16)	
Region						
Northeast	738 (15)	225 (17)	204 (18)	171 (13)	138 (13)	<.001
North Central	1390 (29)	389 (29)	350 (31)	414 (33)	237 (23)	
South	1,823 (38)	499 (37)	401 (35)	468 (37)	455 (44)	
West	814 (17)	231 (17)	175 (15)	215 (17)	193 (19)	
Charlson Comorbidity Index						
None	2901 (61)	842 (63)	710 (63)	740 (58)	609 (60)	.079
Mild	1648 (35)	439 (33)	384 (34)	460 (36)	365 (36)	
Moderate	165 (3.5)	52 (3.9)	27 (2.4)	52 (4.1)	34 (3.3)	
Severe	51 (1.1)	11 (.82)	9 (.8)	16 (1.3)	15 (1.5)	

^a^Data are presented as n (%) for categorical measures and as mean (SD) for continuous variables.

### Costs

Observed all-cause annual healthcare costs per patient increased substantially in the year following diagnosis. **Tables 3 and 4** represent the observed vs modeled mean all-cause healthcare costs per patient per year for individuals diagnosed with EDS, stratified by pre-index, first-year post-diagnosis, and second-year post-diagnosis periods. Observed mean costs rose from $19 487 (n = 4703) during the pre-index period to $29 598 (n = 4764) in Year 1 post-index, before declining to $22 135 (n = 4701) in Year 2. Modeled costs for the full cohort followed a similar trajectory, increasing from $18 708 (SE, $516) pre-index to $28 529 (SE, $776) in Year 1, then decreasing to $21 268 (SE, $685) in Year 2.

**Table 3.** Observed Mean All-Cause Healthcare Costs Among Users^a^

	**Pre-Index**		**1st Year Post**		**2nd Year Post**	
**Variable**	**n**	**Mean (SE)**	**n**	**Mean (SE)**	**n**	**Mean (SE)**
All costs	4703	19 487 (171)	4764	29 598 (257)	4701	22 135 (195)
Inpatient services	343	5398 (302)	585	8293 (307)	366	5611 (313)
Outpatient services	4665	12 847 (96)	4762	18 464 (136)	4662	13 193 (99)
Emergency department	1563	1941 (32)	1684	2453 (38)	1364	1912 (36)
Primary care physician	4504	1399 (07)	4645	1716 (08)	4451	1309 (07)
Specialty physician	3482	1121 (10)	3908	1428 (11)	3244	965 (09)
Facility outpatient procedures	1546	3802 (58)	1877	5471 (69)	1422	3930 (67)
Mental health services	1639	986 (10)	1840	1271 (12)	1726	1149 (11)
Therapy (OT, PT, speech, other)	1937	852 (06)	2370	1262 (08)	1787	756 (06)
Diagnostics	1662	612 (08)	2423	915 (07)	1487	440 (06)
Laboratory	3470	715 (06)	3788	959 (07)	3518	642 (05)
Radiology	2874	1308 (13)	3173	1644 (15)	2572	1203 (14)
Outpatient drugs	924	1250 (44)	1112	1585 (50)	940	2016 (67)
Ancillary/other	2387	701 (12)	2949	1223 (18)	2528	1012 (17)
Pharmacy (Rx)	4254	3409 (38)	4363	4656 (51)	4274	5172 (58)

Abbreviations: OT, occupational therapy; PT, physical therapy; SE, standard error.

^a^Total cohort, N = 4765. Mean cost among users is calculated as the sum of costs for individuals with ≥1 claim divided by the number of individuals with ≥1 claim in that category.

**Table 4.** Modeled All-Cause Healthcare Costs^a^

**Variable**	**Pre-Index, Mean (SE)**	**1st Year Post, Mean (SE)**	**2nd Year Post, Mean (SE)**
All costs	18 708 (516)	28 529 (776)	21 268 (685)
Inpatient services	3303 (298)	6543 (536)	3919 (355)
Outpatient services	12 419 (298)	17 953 (395)	12 789 (346)
Emergency department	1627 (70)	2149 (91)	1602 (75)
Primary care physician	1387 (32)	1711 (34)	1299 (39)
Specialty physician	1047 (28)	1380 (48)	897 (35)
Facility outpatient procedures	3171 (153)	4821 (188)	3280 (142)
Mental health services	925 (65)	1190 (74)	1075 (74)
Therapy (OT, PT, speech, other)	793 (29)	1199 (36)	707 (28)
Diagnostics	528 (21)	869 (26)	387 (17)
Laboratory	658 (24)	912 (33)	600 (23)
Radiology	1175 (36)	1518 (46)	1053 (44)
Outpatient drugs	878 (123)	1236 (147)	1527 (195)
Ancillary/other	603 (43)	1120 (69)	903 (63)
Pharmacy (Rx)	3291 (170)	4531 (233)	5006 (339)

Abbreviations: OT, occupational therapy; PT, physical therapy; SE, standard error.

^a^Total cohort, N = 4765. Modeled mean cost is calculated as the sum of costs for individuals with ≥1 claim statistically modeled over the entire cohort.

**Figure 2.**
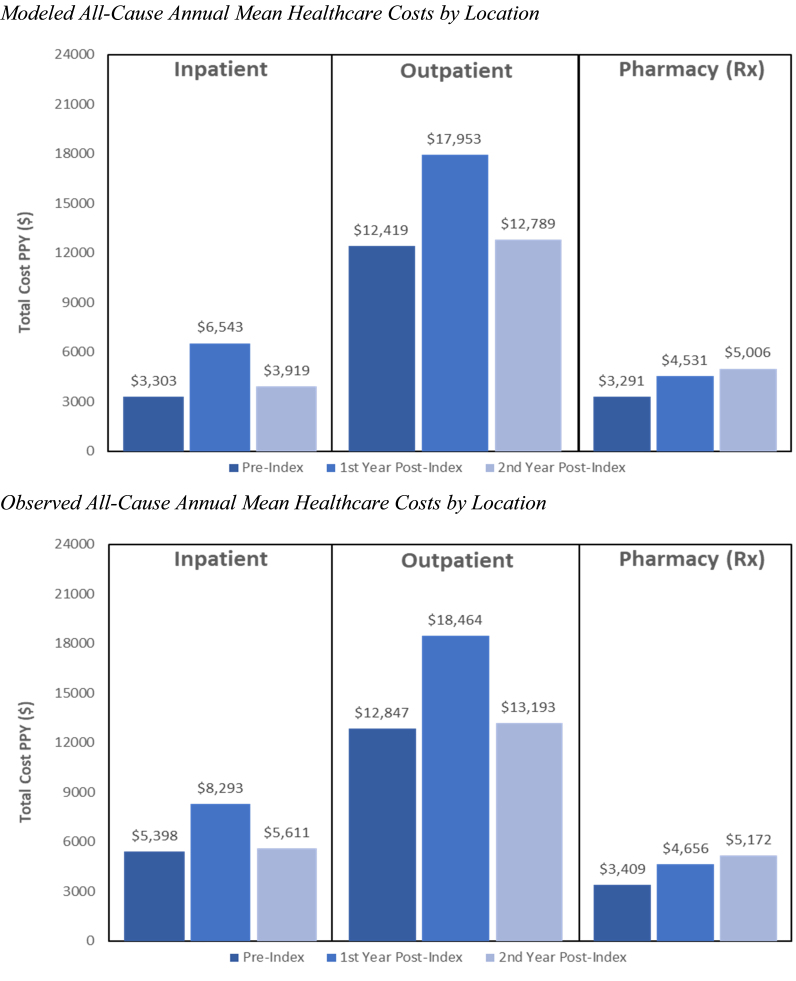
Modeled and Observed All-Cause Annual Mean Healthcare Costs by Location

**Figure 2** shows the annual mean healthcare costs by location. Across categories, inpatient costs among users rose from $5398 (n = 343) pre-index to $8293 (n = 585) in Year 1, before declining to $5611 (n = 366) in Year 2. Outpatient services were the largest contributor to total expenditure, with costs increasing from $12 847 (n = 4665) pre-index to $18 464 (n = 4762) in Year 1, followed by a decline to $13 193 (n = 4662) in Year 2. Within outpatient services, costs for the emergency department ($1941 → $2453 → $1912), primary care ($1399→$1716→$1309), and specialty visits ($1121 → $1428 → $965) followed similar peak-and-decline patterns. Modeled estimates for inpatient, outpatient, and pharmacy services closely mirrored observed results. Pharmacy costs showed sustained growth, increasing from $3409 pre-index to $4656 in Year 1 and $5172 in Year 2.

### Two-Part Models

The two-part model confirmed these descriptive trends. In the binary component, patients were significantly more likely to incur healthcare costs in Year 1 post-index relative to the pre-index baseline (coefficient, 4.155; *P* < .001), while no significant difference was observed for Year 2 (coefficient = –0.033, *P* = .857). Female patients (coefficient, 1.323; *P* < .001) and those with mild comorbidity (coefficient, 1.334; *P* < .001) had higher odds of incurring costs, whereas younger patients (ages 18-34 and 45-54 years) and those residing in the West showed lower odds.

In the gamma model among cost users, both Year 1 (coefficient, 0.412; *P* < .001) and Year 2 (coefficient, 0.129, *P* = .002) were associated with higher expenditures compared with baseline. Female sex was again associated with higher costs (coefficient, 0.463, *P* < .001). A clear comorbidity gradient was observed, with coefficients increasing from mild (0.670, *P* < .001) to moderate (1.321, *P* < .001) and severe (1.935, *P* < .001) CCI levels. Regionally, patients in the North Central and South exhibited significantly lower expenditures compared to the reference region.

## DISCUSSION

This study provides new evidence on the healthcare cost burden associated with EDS using a large, longitudinal claims dataset. Results demonstrate that annual expenditures per patient increased substantially in the first year following diagnosis, followed by a partial decline in the second year, though costs remain above pre-index levels. The consistency between observed and modeled costs strengthened the validity of our findings and underscored that the initial post-diagnosis period is associated with markedly higher expenditures.^16,17^

Outpatient services emerged as the largest contributor to overall costs, accounting for the majority of spending across all time periods. Within outpatient categories, facility-based procedures and emergency department services exhibited notable increases immediately following diagnosis, highlighting the role of diagnostic workups and acute care in early disease management. Although outpatient costs declined in the second year, they did not return to baseline, indicating ongoing healthcare needs. In contrast, pharmacy expenditures showed steady growth across the study period, underscoring the importance of medication management in the chronic care of EDS. Inpatient costs, while less frequent, were substantial when incurred, reflecting the resource intensity of hospitalizations in this population.

The regression models further clarify the drivers of cost burden. Female patients consistently demonstrated higher expenditures, aligning with prior literature suggesting that women with EDS experience more severe symptom profiles and higher care-seeking behaviors.^18–21^ Increasing CCI scores were strongly associated with greater costs, reflecting the cumulative burden of managing both EDS and coexisting health conditions. Regional differences in costs, with lower expenditures observed in the North Central and South regions, suggest potential variation in healthcare access, delivery practices, or pricing structures across geographic markets.

Diagnosing EDS is notoriously complex due to its broad phenotypic variability, nonspecific symptoms, and overlap with other heritable connective-tissue disorders.^2,4–6^ There is currently no definitive biomarker for the most common form, hEDS; diagnosis relies on clinical evaluation using the Beighton scale, family history, and exclusion of other disorders according to the 2017 International Classification criteria.^5^ These diagnostic limitations often lead to prolonged diagnostic journeys, repeated imaging and specialist consultations, and occasional misclassification. Such diagnostic uncertainty likely contributes to the elevated costs observed in the pre-index and early post-index periods of this study. Individuals may accumulate extensive medical expenses before receiving a confirmed EDS diagnosis, reflecting the cost of evaluations across multiple specialties rather than disease progression alone. Similar diagnostic-related cost spikes have been reported in other rare diseases that require complex clinical evaluation, such as muscular dystrophy and cystic fibrosis.^7–9^

The 2017 International Classification of EDS aimed to improve diagnostic precision, but as noted by Malfait et al and Castori et al, substantial overlap remains among subtypes and related syndromes.^5,6,21^ Consequently, administrative claims data may misattribute patients with undiagnosed or misclassified EDS to adjacent diagnostic categories. While the absence of a matched comparator cohort limits causal inference, we intentionally excluded conditions such as Marfan syndrome, muscular dystrophy, and cystic fibrosis because these are among the disorders with which EDS is frequently misdiagnosed.

As Meester et al describe, the clinical overlap among connective tissue syndromes such as Marfan, EDS, and Loeys–Dietz can result in diagnostic substitution, particularly in administrative claims data.^22^ Including these conditions as comparators could therefore introduce contamination, as some individuals coded under these diagnoses may in fact have undiagnosed EDS, thereby biasing comparative cost estimates. Future research should instead incorporate non-overlapping chronic disease comparators or employ matched synthetic-control methods to quantify incremental EDS-specific costs.

These findings extend prior work on the economic burden of rare diseases by quantifying cost trajectories specific to EDS. While prior studies have described the prevalence and clinical heterogeneity of EDS, few have systematically evaluated costs in the period following diagnosis. By documenting both the initial spike in expenditures and the persistence of elevated costs in the second year, this study highlights the need for care models that balance the intensity of early management with strategies for sustainable long-term resource use.

Strengths of this study include the use of a large, nationally representative claims database, robust two-part modeling methods, and the inclusion of both observed and modeled estimates. Limitations include reliance on administrative claims, which may be subject to miscoding and lack clinical detail such as disease severity, laboratory results, or patient-reported outcomes, limiting the ability to assess detailed clinical characteristics of EDS patients.^23^ Additionally, results are limited to insured populations, specifically individuals covered by employer-sponsored health plans and Medicare Supplemental plans. Therefore, findings may not be generalizable to uninsured individuals, Medicaid beneficiaries, or populations with different payer structures.

Finally, while claims data provide comprehensive cost information, they did not capture indirect costs such as lost productivity, caregiver burden, or out-of-pocket expenses that are not reimbursed by insurers. As a result, the study may underestimate the total economic burden of EDS. Despite these limitations, the MarketScan® databases have been widely used in health economics and outcomes research, and their strengths in capturing real-world healthcare utilization and costs outweigh these methodological constraints.^24^

## CONCLUSION

Healthcare costs among patients with EDS rise sharply in the first year following diagnosis and remain elevated in the second year, even after partial declines. Outpatient services and pharmacy expenditures are the primary drivers of spending, while inpatient services contribute disproportionately when hospitalizations occur. Female sex and higher comorbidity burden are consistent predictors of higher costs, and regional variation indicates potential differences in access or care delivery.

This analysis adds to the limited evidence base on the economic burden of EDS by characterizing cost trajectories over time using a large, nationally representative claims database. The results revealed a pronounced peak in total healthcare costs immediately following diagnosis, with costs significantly increasing in the first post-diagnosis year before declining in the second year. This pattern likely reflects the intensity of diagnostic evaluation and early management that accompanies confirmation of EDS. Although total expenditures decreased in the second year, they remained above pre-diagnosis levels, indicating continued healthcare needs and ongoing management demands.

Because this was an observational, claims-based study without a matched comparator cohort, results should be interpreted as descriptive rather than causal. The study was not designed to isolate costs directly attributable to EDS but rather to quantify overall healthcare spending patterns surrounding diagnosis. Future research integrating clinical data or prospective cohorts will be essential to determine incremental EDS-specific costs and to evaluate the effects of care coordination, treatment strategies, and access disparities on long-term outcomes.

These findings highlight opportunities for healthcare planning and policy—specifically, the need to strengthen chronic-disease management, facilitate earlier and more accurate diagnosis, and ensure equitable access to multidisciplinary care. By addressing these factors, healthcare systems can move toward more effective and sustainable models of care for individuals living with EDS.

## Supplementary Material

Online Supplementary Material
