# Supplementary material for: Understanding Ehlers-Danlos Syndrome: A Claims-Based Analysis of Healthcare Cost Patterns
Source: J Health Econ Outcomes Res. 2026 Jul 15;13(2):8–15. doi: 10.36469/001c.145941 (PMC13378745; doi:10.36469/001c.145941)
Supplement: Online Supplementary Material [file jheor_2026_13_2_145941_353805.pdf]

Journal of Health Economics and Outcomes Research

Online Supplementary Material

Understanding Ehlers-Danlos Syndrome: A Claims-Based Analysis of Healthcare Cost Patterns. *JHEOR*. 2026;13(2):8-15. doi:10.36469/jheor.2025.145941

Table S1: Codes Identifying EDS and Related Comorbidities Diagnoses and Relevant Procedure Codes

Table S2: Excluded Codes

Table S3: Charlson Comorbidity Index

Table S4: Two-Part Model Equation

This supplementary material has been provided by the authors to give readers additional information about their work.

This is an open-access article distributed under the terms of the Creative Commons Attribution 4.0 International License (CCBY-4.0). View this license's legal deed at http://creativecommons.org/licenses/by/4.0 and legal code at http://creativecommons.org/licenses/by/4.0/legalcode for more information.

Table S1. Codes Identifying EDS and Related Comorbidities Diagnoses and Relevant Procedure Codes

Code Type	Codes	Description
ICD-10 CM diagnosis codes	Q79.60	Ehlers-Danlos syndrome, unspecified
	Q79.61	Ehlers-Danlos syndrome, classical type
	Q79.62	Ehlers-Danlos syndrome, hypermobility type
	Q79.63	Ehlers-Danlos syndrome, vascular type
	Q79.69	Ehlers-Danlos syndrome, other type

Abbreviation: ICD-10-CM, *International Classification of Diseases, Tenth Revision, Clinical Modification*.

Table S2. Excluded Codes

Code Type	Codes	Description
ICD-10 CM diagnosis codes	C00 – C97	Cancers, metastatic tumors, any malignancy, including lymphoma and leukemia
	Q90 - Q99	Chromosomal abnormalities, not elsewhere classified

Abbreviation: ICD-10-CM, *International Classification of Diseases, Tenth Revision, Clinical Modification*.

Table S3. Charlson Comorbidity Index

Code Type	Condition	Codes
ICD-10 CM diagnosis codes	Chronic heart disease	I05 I06 I07 I08 I10 I20
	Myocardial infarction	I21.x, I22.x, I25.2
	Congestive heart failure	I09.9, I11.0, I13.0, I13.2, I25.5, I42.0, I42.5-I42.9, I43.x, I50.x, P29.0
	Peripheral vascular disease	I70.x, I71.x, I73.1, I73.8, I73.9, I77.1, I79.0, I79.2, K55.1, K55.8, K55.9, Z95.8, Z95.9
	Cerebrovascular disease	G45.x, G46.x, H34.0, I60.x-I69.x
	Dementia	F00.x-F03.x, F05.1, G30.x, G31.1
	Chronic pulmonary disease	I27.8, I27.9, J40.x-J47.x, J60.x-J67.x, J68.4, J70.1, J70.3
	Rheumatic disease	M05.x, M06.x, M31.5, M32.x-M34.x, M35.1, M35.3, M36.0
	Peptic ulcer disease	K25.x-K28.x
	Mild liver disease	B18.x, K70.0-K70.3, K70.9, K71.3-K71.5, K71.7, K73.x, K74.x, K76.0, K76.2-K76.4, K76.8, K76.9, Z94.4
	Diabetes	E10.0, E10.l, E10.2-E10.5, E10.6, E10.8, E10.9, E11.0, E11.1, E11.6, E11.8, E11.9, E12.0, E12.1, E12.6, E12.8, E12.9, E13.0, E13.1, E13.6, E13.8, E13.9, E14.0, E14.1, E14.6, E14.8, E14.9
	Hemiplegia or paraplegia	G04.1, G11.4, G80.1, G80.2, G81.x, G82.x, G83.0-G83.4, G83.9
	Renal disease	I12.0, I13.1, N03.2-N03.7, N05.2-N05.7, N18.x, N19.x, N25.0, Z49.0-Z49.2, Z94.0, Z99.2
	Moderate or severe liver disease	I85.0, I85.9, I86.4, I98.2, K70.4, K71.1, K72.1, K72.9, K76.5, K76.6, K76.7
	AIDS/HIV	B20.x-B22.x, B24.x

Abbreviations: CPT, Current Procedural Terminology; HCPCS, Healthcare Common Procedure Coding System; ICD-10-CM, *International Classification of Diseases, Tenth Revision, Clinical Modification*.

Table S4. Two-Part Model Equation

**Equation**
For a given outcome Y_i (cost or utilization), let D_i be the indicator for a nonzero value:<br>$D_i = \{1, \text{ if } Y_i > 0\} \{0, \text{ if } Y_i = 0\}$
**Part 1: Logistic model (probability of any outcome)**<br>$\text{logit} [\Pr (D_i = 1 \mid X_i)] = \beta_0 + \beta_1 \text{Time}_i + \beta_2 \text{Age}_i + \beta_3 \text{Sex}_i + \beta_4 \text{Region}_i + \beta_5 \text{ ComorbidityIndex}_i$
**Part 2: Continuous/count model**<br>Cost outcomes (using a gamma distribution):<br>$\ln [E (Y_i \mid Y_i > 0, X_i)] = \gamma_0 + \gamma_1 \text{Time}_i + \gamma_2 \text{Age}_i + \gamma_3 \text{ Sex}_i + \gamma_4 \text{Region}_i + \gamma_5 \text{ ComorbidityIndex}_i$<br>Utilization outcomes (using a negative binomial distribution):<br>$\ln [E (Y_i \mid Y_i > 0, X_i)] = \gamma_0 + \gamma_1 \text{Time}_i + \gamma \text{Age}_i + \gamma_3 \text{ Sex}_i + \gamma_4 \text{Region}_i + \gamma_5 \text{ ComorbidityIndex}_i$
**Combined (unconditional) expected outcome:**<br>$E (Y_i \mid X_i) = \Pr (Y_i > 0 \mid X_i) \text{ x } E (Y_i \mid Y_i > 0, X_i)$<br>In these equations, X_i represents the set of covariates (post-index time period, age group, sex, region, and Charlson Comorbidity Index).
